# The impact of income support systems on healthcare quality and functional capacity in workers with low back pain: a realist review protocol

**DOI:** 10.1186/s13643-019-1003-y

**Published:** 2019-04-09

**Authors:** Michael Di Donato, Ross Iles, Tyler Lane, Alex Collie

**Affiliations:** Insurance Work and Health Group, Health Services Division, School of Public Health and Preventive Medicine, Faculty of Medicine Nursing and Health Sciences, Monash University, 553 St Kilda Road, Melbourne, Victoria 3000 Australia

**Keywords:** Low back pain, Workers’ compensation, Income support, Healthcare, Realist review

## Abstract

**Background:**

Low back pain is the greatest contributor to the global burden of disease and can result in work disability. Previous literature has examined the influence of personal factors, the healthcare system, workplace, and income support systems on work disability due to low back pain. Income support systems may also influence healthcare and the workplace, leading to an impact on healthcare quality and functional capacity. However, there has been little insight as to how or in what contexts this influence occurs. This realist review aims to provide an explanation of how and in what contexts income support systems impact the healthcare quality and functional capacity of people who are unable to work due to low back pain.

**Methods:**

Realist reviews are a type of literature review that seek to determine how and in what contexts a social programme such as income support leads to an outcome, rather than simply determining whether or not it works. Five initial theories about how income support systems impact healthcare quality and functional capacity are posited in this protocol. An iterative search of electronic databases for academic literature will be used to acquire and synthesise evidence that may support or refute these initial theories. Grey literature such as policy documents will be identified to characterise income support and healthcare systems and supplement contextual details. Semi-structured interviews with income support, healthcare, and low back pain experts will also be performed to complement literature searching with anecdotal and experiential evidence. At the conclusion of the review, initial theories will be supported or refuted and refined into programme theories that will be explained by evidence in context-mechanism-outcome configurations.

**Discussion:**

Income support and healthcare systems are highly complex and fluid programmes. At the intersection between these systems are those with low back pain. By using realist review methods, we will provide explanatory rather than judgemental findings. The resulting multi-dimensional and contextual understanding of the impact of income support systems on important low back pain outcomes will provide valuable insight for future income support and healthcare policy development.

**Electronic supplementary material:**

The online version of this article (10.1186/s13643-019-1003-y) contains supplementary material, which is available to authorized users.

## Background

### Non-specific low back pain and work

Non-specific low back pain (NSLBP) is a prevalent symptom that cannot be attributed to an explicit pathology or anatomical structure in the lumbar spine [1–4]. NSLBP is the greatest contributor to the global burden of disease, responsible for approximately 60 million years lived with disability (YLDs) [4, 5]. Contemporary evidence suggests some 540 million people globally are likely to have a form of low back pain, and estimates point to a significant 17.3% increase in prevalence between 2005 and 2015, signifying the problem is only growing [5]. The symptomatic course of NSLBP can be acute, sub-acute, or chronic; is usually recurrent; and can result in activity limitation or participation restriction [6–8]. NSLBP contributes the most disability burden to persons of working age [4, 5]. Work disability resulting from NSLBP is a substantial economic burden. Several previous estimates of the direct costs of NSLBP, such as healthcare and rehabilitation, have cited expenditure in excess of billions of dollars. Direct costs are a fraction of the total costs when accounting for indirect costs, such as lost productivity, that can be more than ten times greater [9]. The human cost of work disability due to NSLBP must also be considered. Workers who experience NSLBP have previously reported “a loss of function”, “damaged relationships”, and a “fear of losing their job” [10].

NSLBP is usually self-limiting with a positive prognosis [8]. Nevertheless, many people still seek treatment; approximately 3 in every 100 General Practitioner visits in Australia were for back pain in 2015–2016 [11]. Evidence-based treatments for NSLBP are well established. There are a multitude of primary studies, systematic reviews, clinical practice guidelines, and overall expert consensus regarding treatment [12]. One key recommendation from most guidelines is that those suffering from NSLBP should continue to be active, and, where possible, stay at work [12–14]. Previous literature has established that work is beneficial for health [15], and recent studies have acknowledged this factor when examining NSLBP [16].

Data indicate a substantial proportion of workers may require single or multiple bouts away from work due to functional capacity impairments arising from NSLBP [16]. A worker may engage in an income support system during an absence from work. An income support system provides income support to replace wages lost due to a work absence and in some cases can also fund healthcare for rehabilitation of the NSLBP. Income support systems can also provide other services, such as job finding and return to work services [17], and are typically organised geographically at a national or regional level. Common examples of income support systems include workers’ compensation and disability insurance [18].

Contemporary literature indicates that despite established evidence-based treatment and management of NSLBP, income support systems may influence healthcare quality for those with NSLBP [19, 20]. The existing evidence base about good and poor quality treatment and diagnostics for NSLBP is substantial [12, 14, 21–23]. Evidence suggests good quality healthcare for NSLBP includes a biopsychosocial approach and a focus on encouraging a return to activities such as work and exercise [12, 14, 21–23]. Evidence also identifies specific treatments such as surgery and opioids and diagnostics such as advancing imaging (MRI) as poor quality, with limited benefits relative to higher risks associated with such procedures and, in some cases, detrimental or adverse outcomes [12, 14, 24–28]. Healthcare quality potentially influences another key outcome, functional capacity [24, 26, 27, 29]. Functional capacity describes the ability of an individual to perform activities of daily living and work and may also be directly influenced by income support systems [30]. Literature further indicates contextual factors such as parallel social security policies, socioeconomic environment, and other national and regional differences could contribute to changes in outcomes such as functional capacity and healthcare quality [20, 31–34].

Current evidence exploring factors affecting workers unable to work due to NSLBP fall within the four domains of the Sherbrooke Model of Work Disability [35]: the personal system, workplace system, healthcare system, and legislative system. However, to date, there has been minimal research about the interactions between these systems. We hypothesise that the legislative system (i.e. the income support system) influences the healthcare and workplace systems, which in turn impacts healthcare quality and functional capacity. Research that has examined the influence of income support systems has simply revealed there may be an interaction, with minimal insight as to how or in what contexts the income support system can influence healthcare quality and functional outcomes [30].

### Realist reviews

With these complex and context-dependent possibilities in mind, we have chosen a realist review methodology as the structure of this literature review. A realist review is a type of evidence synthesis designed to examine how complex social programmes such as income support systems work [36]. Realist reviews are based on the philosophy of realism. Realism provides a platform to explain how social phenomena and programmes work by accepting that both tangible reality and social constructs can generate “real-world” outcomes [36–38]. Realist reviews rely on the generative model of causality rather than the successionist model of causality [36]. This argues that rather than causality being inferred simply from “X cause and Y effect” (successionist), causality can only be proposed when there is an understanding of the mechanism that links the events, and the context in which the mechanism generates an outcome (generative) [36]. Realist reviews provide an “explanatory, rather than judgemental focus”, asking, “What works?”, “How does it work?”, and “Who does it work for?”, rather than simply “Does it work?” [36]. By synthesising evidence about context, mechanism, and outcome, realist reviews create context-mechanism-outcome (CMO) configurations; these form the basis of programme theories, which explain how a social programme works.

Previous reviews have utilised this method for similar questions of complex social systems. O’Campo et al. examined whether and how unemployment insurance policies affected poverty and psychological health in various contexts [39]. The authors noted that whilst previous evidence had identified an association between unemployment insurance policies and poverty and psychological health, there was a minimal insight into how or in what contexts these policies were associated with these outcomes [40]. In our case, we argue that the influence of income support systems on quality of healthcare and functional capacity outcomes in those with NSLBP can only be adequately explained by addressing the following research questions:

1. How and in what contexts do income support systems impact healthcare quality for people who are unable to work due to NSLBP?
2. How and in what contexts do income support systems impact the functional capacity of people who are unable to work due to NSLBP?

## Methods

### Theory development

**Fig. 1:**
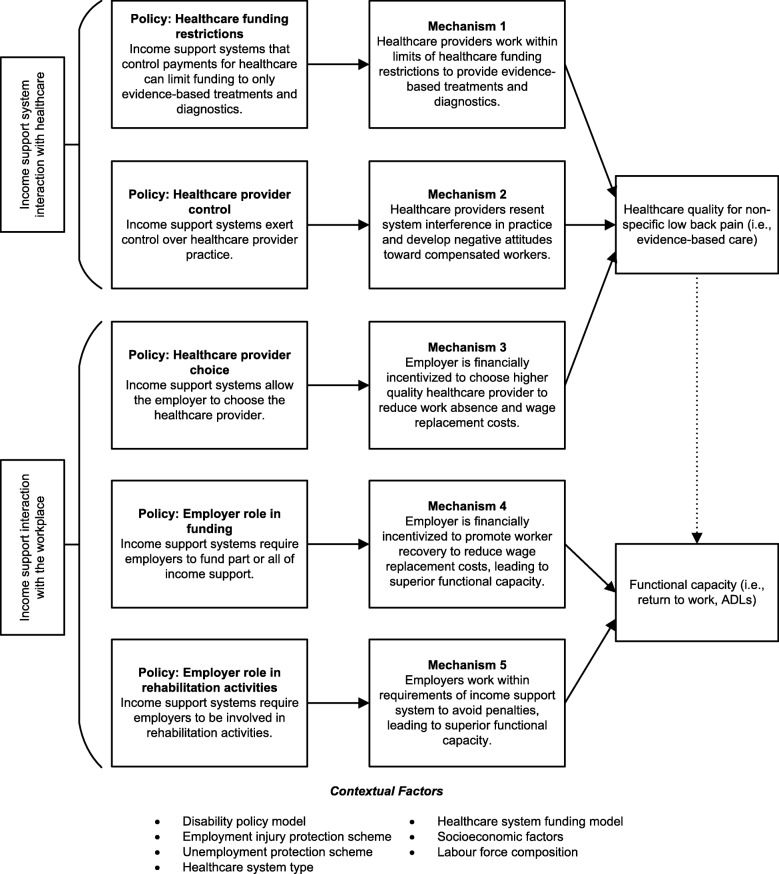
Initial theories

In the first step to addressing these research questions, we conducted initial purposive searching, held several collective author discussions, and consulted experts to develop initial theories that we could test in this review. Our initial theories are programme theories that describe how income support systems may impact healthcare quality and functional capacity. Each initial theory includes a hypothesised mechanism; the “non-observable” process that generates an actual or empirical outcome [37, 41]. We also developed a list of potential contextual factors that may affect how these mechanisms generate outcomes. These initial theories explain the theoretical interactions between the income support and healthcare and workplace systems of the Sherbrooke Model of Work Disability [35]. Our initial theories are described in Fig. 1 (adapted from Nagelhout et al. [42]). Evidence identified during searching, data extraction, and synthesis will be organised and understood in context-mechanism-outcome (CMO) configurations [36]. These CMO configurations will then support or refute our initial theories and underpin explanations of refined programme theories at the conclusion of the review.

As demonstrated in Fig. 1, each mechanism ultimately influences the functional capacity of the worker. Mechanisms may directly influence functional capacity, but we hypothesise that healthcare quality may also affect worker functional capacity; thus, the mechanism may have an indirect influence on functional capacity [24, 26, 27, 29]. Mechanisms 1 and 2 describe how income support systems may interact with the healthcare system to influence healthcare quality [19]. Mechanism 3 describes how income support systems may interact with the workplace to influence healthcare quality [20]. Finally, mechanisms 4 and 5 describe how income support systems interact with the workplace to influence functional capacity [43–45].

Figure 1 also features contextual factors. Context is anything that acts as a barrier, enabler, or manipulator of a mechanism. In our initial theories contextual factors are the characteristics of approaches to income support and healthcare for NSLBP, as well as socioeconomic factors, and labour composition within the region that influences how a mechanism generates an outcome. Characteristics of income support and healthcare systems are contextual features inherent to certain types of income support system. For example, contextual factors in a private healthcare system that relies on the private funding of healthcare services might include the income expectations of a healthcare provider.

**Fig. 2:**
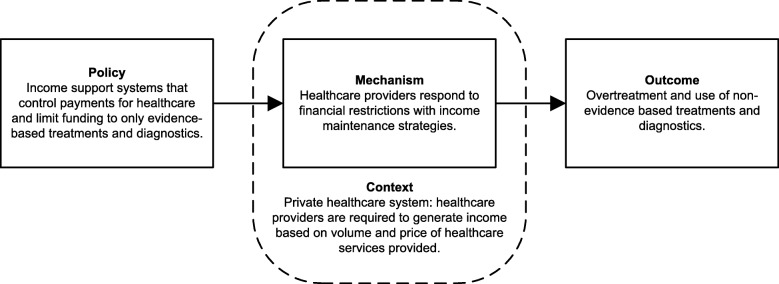
Example of a CMO configuration using an initial theory (mechanism 1)

An example of the distinction between context and mechanism and the role of each is presented in Fig. 2. Here we observe one of our initial theories (mechanism 1) in a CMO configuration using evidence from an American study [20]. The income support system here controls payments for healthcare with a medical fee schedule. This is a policy that could occur in many types of income support systems. However, the characteristics typical of the type of healthcare system modify the mechanism and subsequent outcome.

In the case of Shraim et al. [20], rather than lower medical costs for workers with NSLBP, costs remained similar and the length of disability increased. The authors hypothesised that healthcare providers compensated for the lower fee per procedure by increasing the “volume and complexity” of care to maintain individual income levels (i.e. mechanism). In this case, the private healthcare system (context) perpetuated the need for healthcare providers to maintain income levels (mechanism).

The ability to manipulate healthcare service provision and prices is not inherent to all healthcare systems. The United Kingdom (UK) operates a welfare-state-model healthcare system [46, 47], in which the majority of healthcare providers are government regulated, funded, and owned, substantially reducing the possibility of any perverse market-driven healthcare outcomes [46, 48]. In the context of this type of healthcare system, the same fee schedule policy may not lead to the income maintenance mechanism observed in Shraim et al. [20]. Throughout the review, we have not identified specific characteristics of these systems, instead opting for the overall typologies that may be important.

### Overview

We will adopt several strategies to acquire evidence to sufficiently explain, support, or refute our initial theories. Purposive searching for literature to develop our initial theories was conducted in the development of this protocol. In the review, an iterative search strategy will first be used to “snowball” search results. We will begin by conducting searches of electronic databases for academic literature related to our initial theories. One author (MDD) will conduct these searches to identify literature for the initial theories, and explore the reference lists of identified papers for additional studies. All authors will read and extract data from the identified literature independently, before collectively discussing how extracted data supports or refutes the initial theories, and how data can be organised in CMO configurations and synthesised to refine initial theories into programme theories [49].

**Fig. 3:**
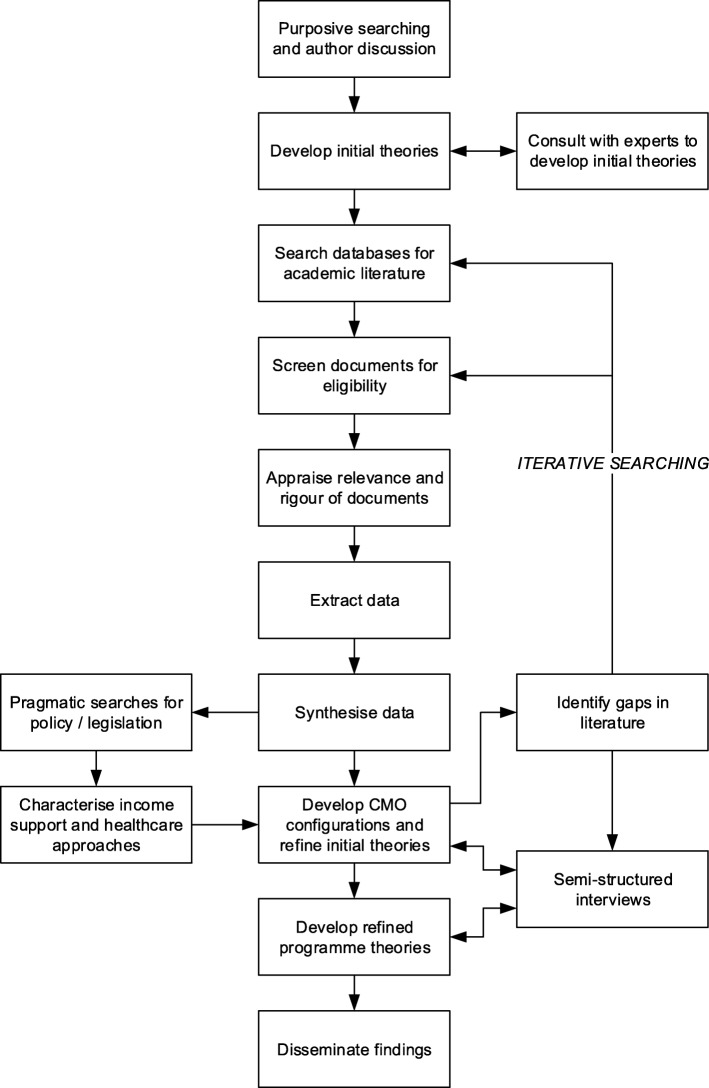
Overall realist review process

Following a round of searching, data extraction, and synthesis, we will collectively judge whether theoretical saturation has been reached, that is, when evidence synthesised from around no longer contributes anything new in the explanation of our refined programme theories [36]. If theoretical saturation is not reached, we will repeat the process until it is judged as such. This will involve using papers identified from the reference lists of papers included from previous searches, before additional searches of electronic databases. Semi-structured interviews with experts will be used in parallel to literature searching to test initial theories and address knowledge gaps that cannot be resolved from further literature searching. Finally, pragmatic searches of policy databases will be performed to identify grey literature that can characterise income support and healthcare approaches for workers with NSLBP that have been identified throughout the previous steps. This process is illustrated in Fig. 3.

### Search strategy

We will conduct searches using key terms in the electronic databases Ovid MEDLINE, Cochrane Library, PsycINFO, and CINAHL. Search terms will include combinations of synonyms based on the terms “non-specific low back pain”, “income support”, “workers compensation”, “social security”, “income protection”, “disability support”, “functional capacity”, “work ability”, “return to work”, “quality of healthcare”, and “medical costs”. These will be combined with the appropriate Boolean operators. If a new database search is required for a new round of searching, we will adjust search terms accordingly.

We will also perform pragmatic searches for grey literature to characterise a country or regions’ approach to income support and healthcare for NSLBP. Grey literature will predominantly consist of policies, policy summaries, and legislation relevant to the country or region. Characterising the concrete boundaries and features of these approaches will contribute to the explanation of both the context and mechanisms in a given region. Understanding the theoretical and legal limits to these approaches will also be useful when interviewing experts; it is possible interviewees could provide insight as to what policies actually reach practice.

We define a region’s income support and healthcare systems by a number of social welfare and healthcare features. These will include the types of unemployment protection scheme [50], disability policy model [51], employment injury protection scheme, healthcare system [46, 48], and healthcare system funding [47] that are legislated and used in a region. A summary of each of these characteristics is presented in Table 1. Further detail regarding each characteristic is available in Additional file 1.

**Table 1:** Income support and healthcare system characteristics (adapted from [46, 48, 50, 51])

Characteristic	Type
Region	Region where the approach is located
Disability policy model	Social-democratic disability policy model, liberal disability policy model, corporatist disability policy model
Employment injury protection scheme type	Social insurance, non-contributory non-means-tested scheme (universal), non-contributory means-tested schemes (social assistance), employer liability
Unemployment protection scheme type	Contributory unemployment benefit schemes, non-contributory unemployment benefit schemes, employment guarantee schemes, unemployment individual savings accounts, severance pay
Healthcare system funding model	Market model, welfare state model, mixed model
Healthcare system type	National health service, national health insurance, social health insurance, private health system, etatist social health insurance
Comments	Additional comments about this approach to supporting workers with NSLBP

We will perform pragmatic searches in select bodies of literature. We selected legislation and policy databases identified and employed by Heymann et al. in a similar study to characterise international sick leave policies [52]. These sources were combined with others and include the International Labour Organisation (ILO) NATLEX, EPLex, LEGOSH, and NORMLEX databases [53–56]; United States Social Security Administration (US-SSA) databases [57]; International Social Security Administration (ISSA) publication database [58]; the Mutual Information System on Social Protection (MISSOC) database [59]; and the Organisation for Economic Co-operation and Development (OECD) document library [60]. Where an approach cannot be sufficiently described by literature in these databases, we will purposively search elsewhere for literature such as annual reports.

We will perform pragmatic searches at two stages in the review. First, when a country or region has been identified in academic literature searching; this will provide a contextual background to where an identified mechanism may occur. Secondly, following data synthesis, to ensure no additional information has been missed.

### Eligibility criteria

One author (MDD) will screen titles and abstracts and full texts for eligibility. An additional author (TL) will screen a random sample of 10% of the titles and abstracts and full texts for consistency. A third author (AC) will adjudicate if there are disagreements between authors about the eligibility of literature. Included literature can focus on any system that provides income support for people with NSLBP. We will accept any empirical study design, including literature reviews. It is likely many types of literature will be needed to form the CMO configurations required to refine our initial theories into refined programme theories. We will therefore also accept opinion pieces and letters. We will exclude literature where the population included either participants not of working age or those where NSLBP is caused by specific pathologies such as trauma. If participants include a mix of those not of working age, or with various or unidentified pathologies, the literature will be excluded. Whilst we will not discriminate literature that examines a particular outcome measure, we have chosen to refine included outcomes for simplicity and feasibility purposes. We will include any outcome related to the quality of healthcare. Functional capacity refers to activity limitations such as activities of daily living (ADLs) and participation restrictions such as work. We will exclude measures of impairment, such as pain, mental or physical health, and costs or expenditure. Full eligibility criteria are detailed in Table 2.

**Table 2:** Eligibility criteria

	Inclusion	Exclusion
Study focus	Any system that provides income support for people with NSLBP (e.g. workers’ compensation, disability support systems)	Systems that do not offer income support systems
Study design	Qualitative description Randomised controlled trial (RCT) Cohort study Case control study Cross-sectional study Mixed-methods study Systematic reviews (and meta-analysis) Literature reviews (including narrative) Opinion piece Letter	
Grey literature	Position statements Policy summaries Legislation	
Study population	Working-age adults in whom NSLBP is the cause of any absence from work	Participants younger than 18 years Participants where NSLBP is caused by specific pathologies (e.g. injuries)
Outcome	Healthcare quality worker functional capacity (e.g. work, activities of daily living)	Pain Physical health Mental health Costs or expenditure

Results of each round of searching will be appraised for eligibility for inclusion. The reference lists of included papers will be scanned for additional literature.

### Data extraction and appraisal

All authors will perform data extraction in pairs following each round of searching. We will record details such as population, sample size, and setting for each piece of literature. We will then extract data regarding our theories. We will perform data extraction with the following questions in mind: “how and in what contexts do income support systems impact the quality of healthcare and functional capacity of people who are unable to work due to NSLBP?”, “what are the mechanisms that generate quality of care or functional outcomes?”, “what contextual factors influence these mechanisms?”, and “who experiences these mechanisms, contexts, and outcomes?”. These questions will guide what data are extracted from the included literature. Extracted data can be any aspect of information from the included literature that contributes to the explanation and refinement of our theories. Data can either support or refute our theories, so long as it contributes to the greater understanding. Data will be extracted using an adapted data extraction tool (see Table 3 [40] in an Excel spreadsheet). This data extraction tool has been piloted on a select number of studies. At the completion of each round of data extraction, all authors will collectively discuss any differences in data extraction. These discussions will be adjudicated by an author not from the pair who extracted the data. During data extraction, all authors will note the relevance and rigour of the literature for extraction. Relevance refers to whether or not literature contains data that adequately address an initial theory. Authors will identify literature as very, moderately, or less relevant (see Table 3) based on the contribution of data to the explanation of an initial theory. Rigour is whether or not the data from literature were generated with “credible and trustworthy” methods [36, 38]. That is, the literature includes transparent methodology (i.e. “trustworthiness”), and the arguments posited by the literature are “plausible and coherent” (i.e. “credible”) [61]. These two dimensions are typical of a realist review and are often used as a form of quality appraisal. A range of literature types are required to achieve the explanatory role of a realist review, and it is considered inappropriate to apply quality assessment tools that may hierarchically downgrade studies based on their design [36].

**Table 3:** General data extraction tool

Author		–
Year		–
Type		Primary study? Review? Grey literature?
Region		USA? Australia?
Relevance	Rating	Very relevant, moderately relevant, or less relevant?
	Justification	Does the study adequately address an initial theory? Does it address a mechanism, context, or outcome?
Rigour	Rating	Very rigorous, moderately rigorous, or less rigorous?
	Justification	Did the authors of this study draw an inference that can make a methodologically credible test of a theory?
Context	Mechanism	1, 2, 3, all..?
	Evidence	How does this contextual factor contribute to an initial theory/CMO configuration?
	Page	Page number
	Author interpretation	How do the authors interpret this result? Does the interpretation contribute to our initial theories?
Mechanism	Mechanism	1, 2, 3, all..?
	Position	Support, refute, other?
	Evidence	What is the evidence for the mechanism proposed by this literature? How and why does it generate an outcome? How does it fit with our initial theories?
	Page	Page number
	Author interpretation	How do the authors interpret this result? Does the interpretation contribute to our initial theories?
Outcome	Evidence	What is the outcome of the mechanism in this literature? How is this outcome defined?
	Location	Page number, paragraph
	Author interpretation	How do the authors interpret this result? Does the interpretation contribute to our initial theories?
Comments		Additional comments about this literature
Citation		Any citations in this article that could be appropriate for inclusion in the review

### Semi-structured interviews

We will hold ten to 15 semi-structured interviews with experts in the fields of income support systems, healthcare, and NSLBP. These will be conducted either face-to-face or via telephone and will occur throughout the review process. Participants will be purposively sampled according to both their role in an income support system or healthcare system and any gaps in academic literature findings. Participants will hold roles in policy, research, or in direct engagement with workers. Interviews will be structured around our initial theories and contextual factors and gaps in academic literature or understanding. In alignment with realist interviewing techniques, participants will be asked whether they agree with our initial theory, and why they do or do not [62]. We expect gaps in literature will centre around the mechanisms and major contextual factors, which are more likely to be supported by anecdotal and experiential evidence, rather than empirical. Interviews will be analysed using realist logic of analysis [62]. Interviewee responses to each initial theory will be summarised and prepared for discussion with identified literature during the synthesis stage of the review. Expert interviewees will not be identified in reporting this review; only their role and experience will be referred to during data reporting and synthesis. We acquired ethics approval from the Monash University Human Research Ethics Committee (Project ID 14144, July 2018). Whilst interviews will provide us with important anecdotal information to fill gaps from literature searching, they are not exhaustive, and we acknowledge that they will only answer part of a more complex picture that may be better answered by a future realist evaluation.

### Data analysis and synthesis

We will perform data synthesis in group discussions with all authors following each round of literature searching, data extraction, and semi-structured interviews. This step will involve situating mechanisms in different contexts, adjudicating evidence based on relevance and rigour, juxtaposing between evidence that presents contrasting outcome patterns, reconciling differences to explain inconsistent outcomes, and consolidating evidence to create CMO configurations that will support or refute our initial theories and underpin the explanations of our refined theories [38, 49]. All evidence, including data extracted from academic literature, findings from semi-structured interviews, and the characteristics of income support and healthcare approaches for workers with NSLBP, will be included in this process.

Author judgements about the relevance and rigour of the included literature made during data extraction will be important in the synthesis stage. We will collectively appraise these judgements to adjudicate between articles and studies. For example, we may grant less weight to less relevant articles in the overall synthesis if there is already sufficient alternative, more relevant literature available after data extraction.

Synthesis is likely to occur multiple times as we move through an iterative methodology. This step in the methods will indicate when we have reached theoretical saturation and can discontinue searching. We will explicitly document our reasoning for each refinement of the programme theories.

### Methodological quality assurance

Realist review methods demand more fluid methodology than systematic reviews; concepts such as an iterative search method, or mixed-methods assessments, leave realist reviews open to methodological criticism. A number of steps have therefore been prepared to ensure that the realist review meets the highest quality standards:

1. All authors will participate in a number of workshops throughout the review process. These workshops will be held when the authors deem sufficient work has occurred since the last workshop to warrant a group discussion for synthesis of results.
2. We will conduct both internal and external methodological quality assessments using Wong et al.’s quality standards for realist reviews [63]. We will refer to these quality standards to ensure that we are performing a rigorous and high-quality review throughout the review process.
3. Whilst we acknowledge that realist reviews are an iterative non-linear process, with all steps subject to change until the review is complete, we developed this protocol to outline our methods a priori, and we will discuss deviations in our methods in the final review.
4. We have completed the PRISMA-P checklist for this protocol [64].

## Discussion

Non-specific low back pain is the greatest contributor to the global burden of disease and results in work disability for the individual and an economic burden for society [4, 5, 9, 65]. Income support and healthcare systems are highly complex and fluid programmes; each is based on intricate policies and relies on the interactions between multiple stakeholders [66]. At the intersection between these systems are people with NSLBP. Interactions between stakeholders, policies, and contextual factors are likely to generate numerous possible outcomes. By using realist review methods, we will provide explanatory rather than judgemental findings [36]. The resulting multi-dimensional and contextual understanding of the impact of income support systems on important NSLBP outcomes will provide valuable insight for future income support and healthcare policy development.

## Additional file

Additional file 1: **Table S1.** Detail of income support and healthcare approach characteristics. (DOCX 50 kb)

## Abbreviations

ADL: Activity of daily living
CMO: Context-mechanism-outcome
ILO: International Labour Organisation
NSLBP: Non-specific low back pain
OECD: Organisation for Economic Co-Operation and Development
US-SSA: United States Social Security Administration

## References

[1] Australian Institute of Health and Welfare (AIHW). Musculoskeletal fact sheet: back problems. Canberra: AIHW; 2015. https://www.aihw.gov.au/getmedia/72d28f53-cf36-40b9-b122-8415de81b1f7/back-problems-factsheet-phe185.pdf.aspx?inline=true. Accessed 8 Feb 2018.

[2] Dunn KM, Hestbaek L, Cassidy JD (2013). Low back pain across the life course. Best Pract Res Clin Rheumatol.

[3] Duthey B. Background paper 6.24 low back pain: World Health Organisation (WHO); 2013. http://www.who.int/medicines/areas/priority_medicines/BP6_24LBP.pdf. Accessed 12 Mar 2018.

[4] Hartvigsen Jan, Hancock Mark J, Kongsted Alice, Louw Quinette, Ferreira Manuela L, Genevay Stéphane, Hoy Damian, Karppinen Jaro, Pransky Glenn, Sieper Joachim, Smeets Rob J, Underwood Martin, Buchbinder Rachelle, Hartvigsen Jan, Cherkin Dan, Foster Nadine E, Maher Chris G, Underwood Martin, van Tulder Maurits, Anema Johannes R, Chou Roger, Cohen Stephen P, Menezes Costa Lucíola, Croft Peter, Ferreira Manuela, Ferreira Paulo H, Fritz Julie M, Genevay Stéphane, Gross Douglas P, Hancock Mark J, Hoy Damian, Karppinen Jaro, Koes Bart W, Kongsted Alice, Louw Quinette, Öberg Birgitta, Peul Wilco C, Pransky Glenn, Schoene Mark, Sieper Joachim, Smeets Rob J, Turner Judith A, Woolf Anthony (2018). What low back pain is and why we need to pay attention. The Lancet.

[5] Vos T (2016). Global, regional, and national incidence, prevalence, and years lived with disability for 310 diseases and injuries, 1990-2015: a systematic analysis for the Global Burden of Disease Study 2015. Lancet (London, England).

[6] Walker BF, Muller R, Grant WD (2003). Low back pain in Australian adults: the economic burden. Asia Pac J Public Health.

[7] Wasiak R, Kim J, Pransky G (2006). Work disability and costs caused by recurrence of low back pain: longer and more costly than in first episodes. Spine.

[8] Young AE, Wasiak R, Gross DP (2013). Recurrence of work-related low back pain and disability: association between self-report and workers’ compensation data. Spine.

[9] Dagenais S, Caro J, Haldeman S (2008). A systematic review of low back pain cost of illness studies in the United States and internationally. Spine J.

[10] Froud R, Patterson S, Eldridge S, Seale C, Pincus T, Rajendran D, Fossum C, Underwood M (2014). A systematic review and meta-synthesis of the impact of low back pain on people’s lives. BMC Musculoskelet Disord.

[11] Australian Institute of Health and Welfare (AIHW). Back problems - what role do GPs play in treating back problems? https://www.aihw.gov.au/reports/arthritis-other-musculoskeletal-conditions/back-problems/what-role-do-gps-play-in-treating-back-problems. Accessed 15 May 2018.

[12] Foster Nadine E, Anema Johannes R, Cherkin Dan, Chou Roger, Cohen Steven P, Gross Douglas P, Ferreira Paulo H, Fritz Julie M, Koes Bart W, Peul Wilco, Turner Judith A, Maher Chris G, Buchbinder Rachelle, Hartvigsen Jan, Cherkin Dan, Foster Nadine E, Maher Chris G, Underwood Martin, van Tulder Maurits, Anema Johannes R, Chou Roger, Cohen Stephen P, Menezes Costa Lucíola, Croft Peter, Ferreira Manuela, Ferreira Paulo H, Fritz Julie M, Genevay Stéphane, Gross Douglas P, Hancock Mark J, Hoy Damian, Karppinen Jaro, Koes Bart W, Kongsted Alice, Louw Quinette, Öberg Birgitta, Peul Wilco C, Pransky Glenn, Schoene Mark, Sieper Joachim, Smeets Rob J, Turner Judith A, Woolf Anthony (2018). Prevention and treatment of low back pain: evidence, challenges, and promising directions. The Lancet.

[13] Almeida M, Saragiotto B, Richards B, Maher CG (2018). Primary care management of non-specific low back pain: key messages from recent clinical guidelines. Med J Aust.

[14] National Institute for Health and Care Excellence (NICE). Low back pain and sciatica in over 16s: assessment and management. London: NICE; 2016. https://www.nice.org.uk/guidance/ng59/resources/low-back-pain-and-sciatica-in-over-16s-assessment-and-management-pdf-1837521693637. Accessed 14 May 2018. 27929617

[15] Waddell G, Burton K (2006). Is work good for your health and well-being?.

[16] Cote P, Baldwin ML, Johnson WG, Frank JW, Butler RJ (2008). Patterns of sick-leave and health outcomes in injured workers with back pain. Eur Spine J.

[17] Collie A, Iles R, Di Donato M (2018). The cross sector project: mapping Australian systems of income support for people with health related work incapacity.

[18] Lippel K, Lötters F, Loisel P, Anema JR (2013). Public insurance systems**:** a comparison of cause-based and disability-based income support systems. Handbook of work disability: prevention and management.

[19] Kilgour E, Kosny A, McKenzie D, Collie A (2015). Healing or harming? Healthcare provider interactions with injured workers and insurers in workers’ compensation systems. J Occup Rehabil.

[20] Shraim M, Cifuentes M, Willetts JL, Marucci-Wellman HR, Pransky G (2015). Length of disability and medical costs in low Back pain: do state workers’ compensation policies make a difference?. J Occup Environ Med.

[21] Agency for Clinical Innovation (ACI) (2016). Management of people with acute low back pain.

[22] Qaseem A, Wilt TJ, McLean RM, Forciea MA (2017). Noninvasive treatments for acute, subacute, and chronic low back pain: a clinical practice guideline from the American College of Physicians. Ann Intern Med.

[23] Stochkendahl Mette Jensen, Kjaer Per, Hartvigsen Jan, Kongsted Alice, Aaboe Jens, Andersen Margrethe, Andersen Mikkel Ø., Fournier Gilles, Højgaard Betina, Jensen Martin Bach, Jensen Lone Donbæk, Karbo Ture, Kirkeskov Lilli, Melbye Martin, Morsel-Carlsen Lone, Nordsteen Jan, Palsson Thorvaldur Skuli, Rasti Zoreh, Silbye Peter Frost, Steiness Morten Zebitz, Tarp Simon, Vaagholt Morten (2017). National Clinical Guidelines for non-surgical treatment of patients with recent onset low back pain or lumbar radiculopathy. European Spine Journal.

[24] Graves JM, Fulton-Kehoe D, Jarvik JG, Franklin GM (2014). Health care utilization and costs associated with adherence to clinical practice guidelines for early magnetic resonance imaging among workers with acute occupational low back pain. Health Serv Res.

[25] Harris IA, Traeger A, Stanford R, Maher CG, Buchbinder R (2018). Lumbar spine fusion: what is the evidence?. Intern Med J.

[26] Webster BS, Bauer AZ, Choi Y, Cifuentes M, Pransky GS (2013). Iatrogenic consequences of early magnetic resonance imaging in acute, work-related, disabling low back pain. Spine.

[27] Webster BS, Choi Y, Bauer AZ, Cifuentes M, Pransky G (2014). The cascade of medical services and associated longitudinal costs due to nonadherent magnetic resonance imaging for low back pain. Spine.

[28] Webster BS, Cifuentes M (2010). Relationship of early magnetic resonance imaging for work-related acute low back pain with disability and medical utilization outcomes. J Occup Environ Med.

[29] Collie Alex, Newnam Sharon, Keleher Helen, Petersen Alan, Kosny Agnieszka, Vogel Adam P., Thompson Jason (2018). Recovery Within Injury Compensation Schemes: A System Mapping Study. Journal of Occupational Rehabilitation.

[30] Bartys S, Frederiksen P, Bendix T, Burton K (2017). System influences on work disability due to low back pain: an international evidence synthesis. Health policy (Amsterdam, Netherlands).

[31] Anema JR, Schellart AJ, Cassidy JD, Loisel P, Veerman TJ, van der Beek AJ (2009). Can cross country differences in return-to-work after chronic occupational back pain be explained? An exploratory analysis on disability policies in a six country cohort study. J Occup Rehabil.

[32] Collie Alex, Lane Tyler J, Hassani-Mahmooei Behrooz, Thompson Jason, McLeod Chris (2016). Does time off work after injury vary by jurisdiction? A comparative study of eight Australian workers' compensation systems. BMJ Open.

[33] Shraim M, Cifuentes M, Willetts JL, Marucci-Wellman HR, Pransky G (2017). Regional socioeconomic disparities in outcomes for workers with low back pain in the United States. Am J Ind Med.

[34] Volinn E, Nishikitani M, Volinn W, Nakamura Y, Yano E (2005). Back pain claim rates in Japan and the United States: framing the puzzle. Spine.

[35] Loisel P, Buchbinder R, Hazard R, Keller R, Scheel I, van Tulder M, Webster B (2005). Prevention of work disability due to musculoskeletal disorders: the challenge of implementing evidence. J Occup Rehabil.

[36] Pawson R, Greenhalgh T, Harvey G, Walshe K (2005). Realist review--a new method of systematic review designed for complex policy interventions. J Health Serv Res Policy.

[37] Westhorp G (2014). Realist impact evaluation: an introduction. ODI Research and Policy in Development.

[38] Wong G, Westhorp G, Pawson R, Greenhalgh T (2013). Realist synthesis - RAMESES training materials.

[39] O'Campo P, Molnar A, Ng E, Renahy E, Mitchell C, Shankardass K, St. John A, Bambra C, Muntaner C (2015). Social welfare matters: a realist review of when, how, and why unemployment insurance impacts poverty and health. Soc Sci Med.

[40] Molnar A, O’Campo P, Ng E, Mitchell C, Muntaner C, Renahy E, St. John A, Shankardass K (2015). Protocol: realist synthesis of the impact of unemployment insurance policies on poverty and health. Eval Program Plann.

[41] Westhorp G, Emmel N (2018). Understanding mechanisms in realist evaluation and research. Doing realist research.

[42] Nagelhout GE, Hummel K, de Goeij MCM, de Vries H, Kaner E, Lemmens P (2017). How economic recessions and unemployment affect illegal drug use: a systematic realist literature review. Int J Drug Policy.

[43] Burkhauser RV, Daly MC, de Jong PR (2008). Curing the Dutch disease: lessons for United States disability policy.

[44] Organisation for Economic Co-operation and Development (OECD) (2007). Sickness and disability schemes in the Netherlands.

[45] Safe Work Australia (SWA) (2017). Comparison of workers' compensation arrangements in Australian and New Zealand 2017.

[46] Bohm K, Schmid A, Gotze R, Landwher C, Rothgang H (2012). Classifying OECD healthcare systems: a deductive approach.

[47] Keleher H, Reynolds L, Willis E. Understanding the Australian health care system, 3rd edn: Chatswood, Elsevier; 2016.

[48] Bohm K, Schmid A, Gotze R, Landwehr C, Rothgang H (2013). Five types of OECD healthcare systems: empirical results of a deductive classification. Health policy (Amsterdam, Netherlands).

[49] Pawson R (2006). Evidence-based policy: a realist perspective.

[50] International Labour Organization (ILO). World Social Protection Report 2017–19 - universal social protection to achieve the sustainable development goals: ILO, Geneva. http://www.ilo.org/wcmsp5/groups/public/%2D%2D-dgreports/%2D%2D-dcomm/%2D%2D-publ/documents/publication/wcms_604882.pdf

[51] Organisation for Economic Co-operation and Development (OECD) (2010). Sickness, disability and work: breaking the barriers.

[52] Heymann J, Rho HJ, Schmitt J, Earle A (2010). Ensuring a healthy and productive workforce: comparing the generosity of paid sick day and sick leave policies in 22 countries. Int J Health Serv.

[53] International Labour Organization (ILO). Database of national labour, social security and related human rights legislation (NATLEX). http://www.ilo.org/dyn/natlex/natlex4.home?p_lang=en. Accessed 14 May 2018.

[54] International Labour Organization (ILO). Employment protection legislation database (EPLex). http://www.ilo.org/dyn/eplex/termmain.home. Accessed 14 May 2018.

[55] International Labour Organization (ILO). Occupational safety and health (LEGOSH). http://www.ilo.org/dyn/legosh/en/f?p=14100:1000:0::NO. Accessed 14 May 2018.

[56] International Labour Organization (ILO). Information system on international labour standards (NORMLEX). http://www.ilo.org/dyn/normlex/en/f?p=NORMLEXPUB:1:0::NO. Accessed 14 May 2018.

[57] United States of America Social Security Administration (SSA). Social security programs throughout the world. https://www.ssa.gov/policy/docs/progdesc/ssptw/. Accessed 5 Feb 2018.

[58] International Social Security Association (ISSA). ISSA Homepage. https://www.issa.int/en. Accessed 14 May 2018.

[59] The European Commission. Mutual information system on social protection (MISSOC). https://www.missoc.org/. Accessed 15 June 2018.

[60] Organisation for Economic Co-operation and Development (OECD). OECD iLibrary**.** http://www.oecd-ilibrary.org/. Accessed 14 May 2018 2018.

[61] Wong G, Emmel N (2018). Data gathering in realist reviews. Doing realist research.

[62] Manzano A (2016). The craft of interviewing in realist evaluation. Evaluation.

[63] Wong G, Greenhalgh T, Westhorp G, Pawson R. RAMESES project quality standards for realist synthesis. London; 2014. http://www.ramesesproject.org/media/RS_qual_standards_researchers.pdf. Accessed 11 Apr 2018.

[64] Moher D, Shamseer L, Clarke M, Ghersi D, Liberati A, Petticrew M, Shekelle P, Stewart LA (2015). Preferred reporting items for systematic review and meta-analysis protocols (PRISMA-P) 2015 statement. Systematic Reviews.

[65] Hoy D, Bain C, Williams G, March L, Brooks P, Blyth F, Woolf A, Vos T, Buchbinder R (2012). A systematic review of the global prevalence of low back pain. Arthritis Rheum.

[66] Braithwaite J. Changing how we think about healthcare improvement. BMJ. 2018;361:k2014. 10.1136/bmj.k2014 PMC5956926 29773537

