# Supplementary material for: The impact of income support systems on healthcare quality and functional capacity in workers with low back pain: a realist review protocol
Source: Syst Rev. 2019 Apr 9;8:92. doi: 10.1186/s13643-019-1003-y (PMC6454741; doi:10.1186/s13643-019-1003-y)
Supplement: Supplementary file 1 — Table S1. Detail of income support and healthcare approach characteristics. (DOCX 50 kb) [file 13643_2019_1003_MOESM1_ESM.docx]

# The impact of income support systems on healthcare quality and functional capacity in workers with low back pain: a realist review protocol

## Authors

Mr Michael Di Donato*, Insurance Work and Health Group, School of Public Health and Preventive Medicine, Health Services Division, Faculty of Medicine Nursing and Health Sciences, Monash University, 553 St Kilda Road, Melbourne, Victoria 3004, Australia

[michael.didonato@monash.edu](mailto:michael.didonato@monash.edu); +61 (0) 3 9905 6417

Dr Ross Iles, Insurance Work and Health Group, School of Public Health and Preventive Medicine, Health Services Division, Faculty of Medicine Nursing and Health Sciences, Monash University, 553 St Kilda Road, Melbourne, Victoria 3004, Australia

[ross.iles@monash.edu](mailto:ross.iles@monash.edu)

Dr Tyler Lane, Insurance Work and Health Group, School of Public Health and Preventive Medicine, Health Services Division, Faculty of Medicine Nursing and Health Sciences, Monash University, 553 St Kilda Road, Melbourne, Victoria 3004, Australia

[tyler.lane@monash.edu](mailto:tyler.lane@monash.edu)

Professor Alex Collie, Insurance Work and Health Group, School of Public Health and Preventive Medicine, Health Services Division, Faculty of Medicine Nursing and Health Sciences, Monash University, 553 St Kilda Road, Melbourne, Victoria 3004, Australia

[alex.collie@monash.edu](mailto:alex.collie@monash.edu)

*Corresponding author

# Additional file 1

Table 4 Detail of income support and healthcare approach characteristics (adapted from [1-4])

| Characteristic | Type | Definition |
| --- | --- | --- |
| Region | Region where the approach is located | - |
| Disability policy model | Social-democratic disability policy model | Generous and accessible compensation policy with mostly universal benefit coverage and low benefit entry thresholds |
|  | Liberal disability policy model | Less generous compensation policy with lower benefit levels and higher benefit entry thresholds, including work capacity assessment |
|  | Corporatist disability policy model | Benefits relatively accessible and generous, with developed employment programmes and vocational rehabilitation |
| Employment injury protection scheme type | Social insurance | Social protection scheme funded by contributions. |
|  | Non-contributory non-means-tested scheme (universal) | Social protection scheme that does not require contribution, with no means-testing for eligibility |
|  | Non-contributory means-tested schemes (social assistance) | Social protection scheme that does not require contribution, but requires means-testing to eligibility |
|  | Employer liability | Social insurance based on contributions from employers at a standardised rate |
| Unemployment protection scheme type | Contributory employment benefit schemes | Social insurance based on collective financing and pooling of unemployment risk |
|  | Non-contributory unemployment benefit schemes | Funded by general taxation, provide lower level of benefits than insurance schemes |
|  | Employment guarantee schemes | Legal entitlement to employment in public works to poor workers |
|  | Unemployment individual savings accounts | Workers accumulate savings in individual accounts, providing income stream if necessary |
|  | Severance pay | Income if a worker is dismissed from their job |
| Healthcare system funding model | Market-model | Private funding with competitive dimension |
|  | Welfare-state model | Government funding with no competitive dimension |
|  | Mixed-model | Mixed funding with moderate competitive dimension |
| Healthcare system type | National health service | State regulated, funded, and provided |
|  | National health insurance | State regulated, funded, but delivered by non-profit providers |
|  | Social health insurance | Societal actors regulated and fund, with private actors providing |
|  | Private health system | Privately regulated, funded, and provided |
|  | Etatist social health insurance | State regulated, societal actors fund, and private actors provide |
| Comments | Additional comments about this approach to supporting workers with NSLBP | - |

# References

1. Bohm K, Schmid A, Gotze R, Landwehr C, Rothgang H. **Five types of OECD healthcare systems: empirical results of a deductive classification**. *Health policy (Amsterdam, Netherlands)* 2013, **113**(3):258-269.

2. Bohm K, Schmid A, Gotze R, Landwher C, Rothgang H. **Classifying OECD healthcare systems: a deductive approach**. Wandel, Bremen; 2012. Available from: <https://www.econstor.eu/bitstream/10419/64809/1/726547131.pdf>. Accessed 9 May 2018.

3. International Labour Organization (ILO). **World Social Protection Report 2017-19 - Universal social protection to achieve the Sustainable Development Goals**. Geneva, Switzerland; 2017. Available from: <http://www.ilo.org/wcmsp5/groups/public/---dgreports/---dcomm/---publ/documents/publication/wcms_604882.pdf>. Accessed 14 May 2018.

4. Organisation for Economic Co-operation and Development (OECD). **Sickness, Disability and Work: Breaking the Barriers**. France; 2010. Available from: <http://www.oecd.org/publications/sickness-disability-and-work-breaking-the-barriers-9789264088856-en.htm>. Accessed 18 June 2018.
